# High-protein diets and testosterone

**DOI:** 10.1177/02601060221132922

**Published:** 2022-10-20

**Authors:** Joseph Whittaker

**Affiliations:** The School of Allied Health and Community, 8709 University of Worcester, UK

**Keywords:** Protein, protein intake, high-protein diet, testosterone, androgens, men

## Abstract

A recent meta-analysis found low-carbohydrate, high-protein diets (> 3.4 g/kg of bodyweight/day) (g/kg/day) decreased men's total testosterone (∼5.23 nmol/L) [Whittaker and Harris (2022) Low-carbohydrate diets and men's cortisol and testosterone: systematic review and meta-analysis. *Nutrition and Health*. DOI: 10.1177/02601060221083079]. This finding has generated substantial discussion, however, it has often lacked clarity and context, with the term ‘high-protein’ being used unqualified. Firstly, diets < 3.4 g/kg/day are not associated with a consistent decrease in testosterone. Secondly, the average protein intake is ∼1.3 g/kg/day, conventional ‘high-protein’ diets are ∼1.8–3 g/kg/day and the vast majority of athletes are < 3.4 g/kg/day; meaning very few individuals will ever surpass 3.4 g/kg/day. To avoid such confusion in the future, the following definitions are proposed: very high (> 3.4 g/kg/day), high (1.9–3.4 g/kg/day), moderate (1.25–1.9 g/kg/day) and low (<1.25 g/kg/day). Using these, very high-protein diets (> 3.4 g/kg/day) appear to decrease testosterone, however high- and moderate-protein diets (1.25–3.4 g/kg/day) do not.

## Introduction

High-protein diets have a number of benefits including increased satiety, weight loss and greater preservation of lean mass during caloric deficits (Leidy et al., 2015). However, a recently published meta-analysis found that low-carbohydrate, high-protein diets (> 3.4 g/kg of bodyweight/day) (g/kg/day) caused a large decrease in men's total testosterone (TT) (∼5.23 nmol/L) (Whittaker and Harris, 2022). This finding has ignited considerable discussion online and in the media (Altmetric, 2022); although the accuracy of such coverage has varied greatly, arguably leading to more confusion than clarity. The primary issue is that the findings of the meta-analysis have not been appropriately contextualised. ‘High-protein’ has no standard definition relating to the amount of dietary protein (Westerterp-Plantenga, 2007), leaving it open to interpretation when used in isolation. Moreover, without reference to average and extreme protein intakes, even quoting a precise figure is difficult to interpret. The interest surrounding this finding also provides an opportunity for a more detailed discussion on the effects of protein intake on testosterone, particularly as this has been somewhat neglected by the literature. Thus, the aims of this commentary are: (1) to clarify and contextualise the meta-analysis’ findings, and (2) to explore additional evidence regarding protein intake and testosterone.

## Protein intake terminology and definitions

Protein intake is commonly measured in three ways: g/kg/day, g/day and percentage of total energy intake (TEI). This article shall use g/kg/day throughout, to maintain consistency. The conversion into other measurements is given in Table 1, along with a reference to common diets and population intakes, for ease of interpretation. Of particular note, is that if one maintains a constant absolute protein intake, but reduces their energy intake, protein as measured as a percentage of TEI seemingly increases. The terms high-, moderate-, and low-protein diet have no unified definition, and thus without this, remain inherently subjective. It is advisable to use numerical figures where possible, but this is not always practical or suitable; for instance in the case of titles and keywords. Therefore, one cannot do away with such terms, but efforts should be made to define them. Similar efforts have been made in regard to low-carbohydrate diets (Noakes and Windt, 2017), which also suffer from a plethora of definitions.

**Table 1.** Definitions of protein intake terminology.

Terminology	g/kg/day	g/day (70 kg individual)	% of TEI (weight-maintaining diet)	% of TEI (weight loss diet, 20% calorie deficit)	Common diets and populations^a^
Very high	> 3.4	> 238	> 38	> 47	
High	1.9–3.4	133–238	21–38	27–47	Paleo diet, zone diet, carnivore diet, bodybuilders, athletes
Moderate	1.25–1.9	88–133	14–21	18–27	General population, Mediterranean diet, vegetarian diet, ketogenic diet, low-carbohydrate diet
Low	< 1.25	< 88	< 14	< 18	Vegan diet

g/kg/day: g/kg of bodyweight/day; TEI: total energy intake.

^a^ These are average estimates made using the author's judgement, given only to aid interpretation. Diets may span several protein intake bands, for instance, one could eat a high-protein vegan diet, but typically they are low in protein.

The proposed definition for a very high-protein diet is above the tolerable upper intake. Although this has not yet been established, the literature enables one to make a partial assessment. Amino acids contain nitrogen, which if not incorporated into the body's structure, is converted into urea to be safely excreted. The urea cycle has a maximal rate of urea synthesis (MRUS), beyond which ammonia may build up, leading to toxic effects; or gastrointestinal function altered to reduce protein absorption (e.g. diarrhoea, delayed gastric emptying) (Bilsborough and Mann, 2006). The MRUS varies by individual and bodyweight, with the lowest value previously estimated at 3.35 g/kg/day, including 0.8 g/kg/day for structural purposes (Bilsborough and Mann, 2006). Interestingly, an 8-week study using a 4.4 g/kg/day protein diet, led to 10/30 participants dropping out versus 0/10 in the control group,^1^ and ‘a few’ complaining of gastrointestinal distress and feeling hot (Antonio et al., 2014). In contrast, diets ∼3.4 g/kg/day appear to be well-tolerated by athletic individuals (Antonio et al., 2015, 2016); altogether suggesting up to 3.4 g/kg/day appears safe (at least in healthy, athletic populations), but beyond that is uncertain.

The proposed definition for a low-protein diet is below what is needed for basic physiological function. The USA dietary guidelines set this at 0.8 g/kg/day, to cover the needs of 97.5% of adults (recommended daily allowance), based on nitrogen balance studies (Institute of Medicine, 2006). However, newer indicator amino acid oxidation studies have estimated this at ∼1.25 g/kg/day, which is similar to population intakes (Fulgoni, 2008; Humayun et al., 2007; Rafii et al., 2015a, 2015b). Thus, taking the higher, and therefore more conservative estimate, a low-protein diet would be < 1.25 g/kg/day. The proposed definition for a high-protein diet is an intake sufficient for the average athlete (estimated average requirement). Indicator amino acid oxidation studies estimate this at ∼1.9 g/kg/day, which is again higher than nitrogen balance studies, and more similar to athlete population intakes (Bandegan et al., 2017, 2019; Jenner et al., 2019; Tarnopolsky et al., 1988). Interestingly, this is at the lower end of the 20th-century hunter-gatherer diets (Cordain et al., 2000), which by modern standards are an athletic population. The definitions and bands are listed in Table 1, to add further clarity.

## Protein intake and testosterone

### Intervention studies

The meta-analysis in question found that diets > 3.4 g/kg/day protein decreased TT, a finding that was based on three studies, which are listed at the top of Table 2 (Whittaker and Harris, 2022). Protein intakes > 3.4 g/kg/day are above the general population's, commonly used diets, and the vast majority of athletes (Table 1). The meta-analysis found no consistent effect on TT of diets <3.4 g/kg/day protein. To elucidate this, Table 2 lists the five other studies from the meta-analysis with a substantial difference in protein intake, along with three additional studies with a substantial difference in protein intake, in the latter case, achieved by supplementation (protein vs. carbohydrate). The three additional studies also have higher carbohydrate intakes (41 – 46% of TEI), which helps to elucidate the effects of increasing protein intake, within a moderate-carbohydrate diet; something which the meta-analysis did not examine. The complete set of studies within Table 2, again illustrates that whilst diets > 3.4 g/kg/day consistently decrease TT, diets < 3.4 g/kg/day show no consistent effect on TT. Importantly, the three > 3.4 g/kg/day studies had fairly diverse carbohydrate intakes (5–35% of TEI), but similar protein intakes (3.5 – 3.9 g/kg/day); indicating that protein, not carbohydrate was responsible for the decrease in TT. Similarly, the meta-analysis found no consistent effect on TT of low-carbohydrate diets, < 3.4 g/day/kg protein; again indicating no effect of carbohydrate intake on TT. Lastly, although the three studies > 3.4 g/day/kg found a consistent decrease in TT, they had a small total sample size (*n* = 26), limiting the strength of evidence, and ranged from 3 to 10 days, meaning the long-term effects of such diets are relatively unknown.

**Table 2.** Intervention studies on protein intake and testosterone.^a^

Study	Study type	Sample size	Duration of diet 1 (days)	Macronutrients, % of TEI (protein/fat/carbohydrate)		Protein intake (g/kg/day)		Change in total testosterone (diet 1 vs. 2)^b^	
				Diet 1	Diet 2	Diet 1	Diet 2	nmol/L	%
Anderson et al., 1987	Crossover	7	10	44/21/35	10/20/70	3.9	0.9	−3.36	−20.7
Jaffe, 2013	Crossover	10	7	55/39/7	25/20/55	3.7	1.8	−14.46	−29.8
Langfort et al., 2001	Crossover	9	3	45/50/5	20/30/50	3.5	1.6	−3.44	−15.5
Michalczyk et al., 2019	Crossover	15	28	31/59/10	15/31/54	3.2	1.5	+ 3.32	+ 17.5
Toma, 2009	Parallel	17	49	30/39/31	14/25/58	2.6	1.1	−4.23	−19.4
Pourabbas et al., 2021 ^c^	Parallel	30	42	25/30/45	17/32/50	2.3	1.4	+ 0.49	+ 1.6
Volek et al., 2002	Parallel	20	42	30/61/8	16/26/58	2.2	0.9	−0.2	−1
Tsai et al., 1993 ^d^	Crossover	4	3	25/60/15	15/30/55	2.2	1.3	+ 3.2	+ 14.2
Hoffman et al., 2007 ^c^	Parallel	21	84	25/30/46	15/29/56	2	1.2	−0.73	−2.6
Haun et al., 2018 ^c^^,^^e^	Parallel	32	84	24/35/41	16/35/50	1.8	1.3	+ 5.48	+ 25.4

g/kg/day: g/kg of bodyweight/day; TEI: total energy intake.

^a^ Diets one and two were isocaloric, defined as within 10% of the total energy intake of each other, taking into account differences in energy intake requirements between groups. All samples were healthy, non-obese, young to middle-aged men.

^b^ Change from baseline scores used where possible, and in these studies, the percentage change in testosterone was calculated using the starting value for diet one (Haun et al., 2018; Hoffman et al., 2007; Jaffe, 2013; Pourabbas et al., 2021; Toma, 2009; Volek et al., 2002).

^c^ These studies used a protein vs carbohydrate supplement intervention, to increase protein intake. The unmarked studies used a higher protein diet; although often including protein supplements as well.

^d^ ‘F’ and ‘CHO’ diets were used.

^e^ ‘PLA’ and ‘WPC’ diets were used.

Regarding the testosterone response to exercise, one of the three > 3.4 g/kg/day studies measured this, finding a decrease in during- and post-exercise TT (∼16%, 3.92 nmol/L; 16.6%, 4.86 nmol/L) (Langfort et al., 2001). Supporting this, a small observational study (*n* = 10) found that protein intake, post-exercise TT and free testosterone were inversely correlated (*r* = −0.86, *r* = −0.65) (Sallinen et al., 2004). Two studies using low-carbohydrate diets with < 3.4 g/kg/day protein, found no decrease in post-exercise TT, but one found a non-significant decrease in during-exercise TT (Durkalec-Michalski et al., 2021; Zajac et al., 2014); altogether suggesting that protein > 3.4 g/kg/day may decrease the testosterone response to exercise, but the effects of carbohydrate are unclear.

### Observational research

**Figure 1.**
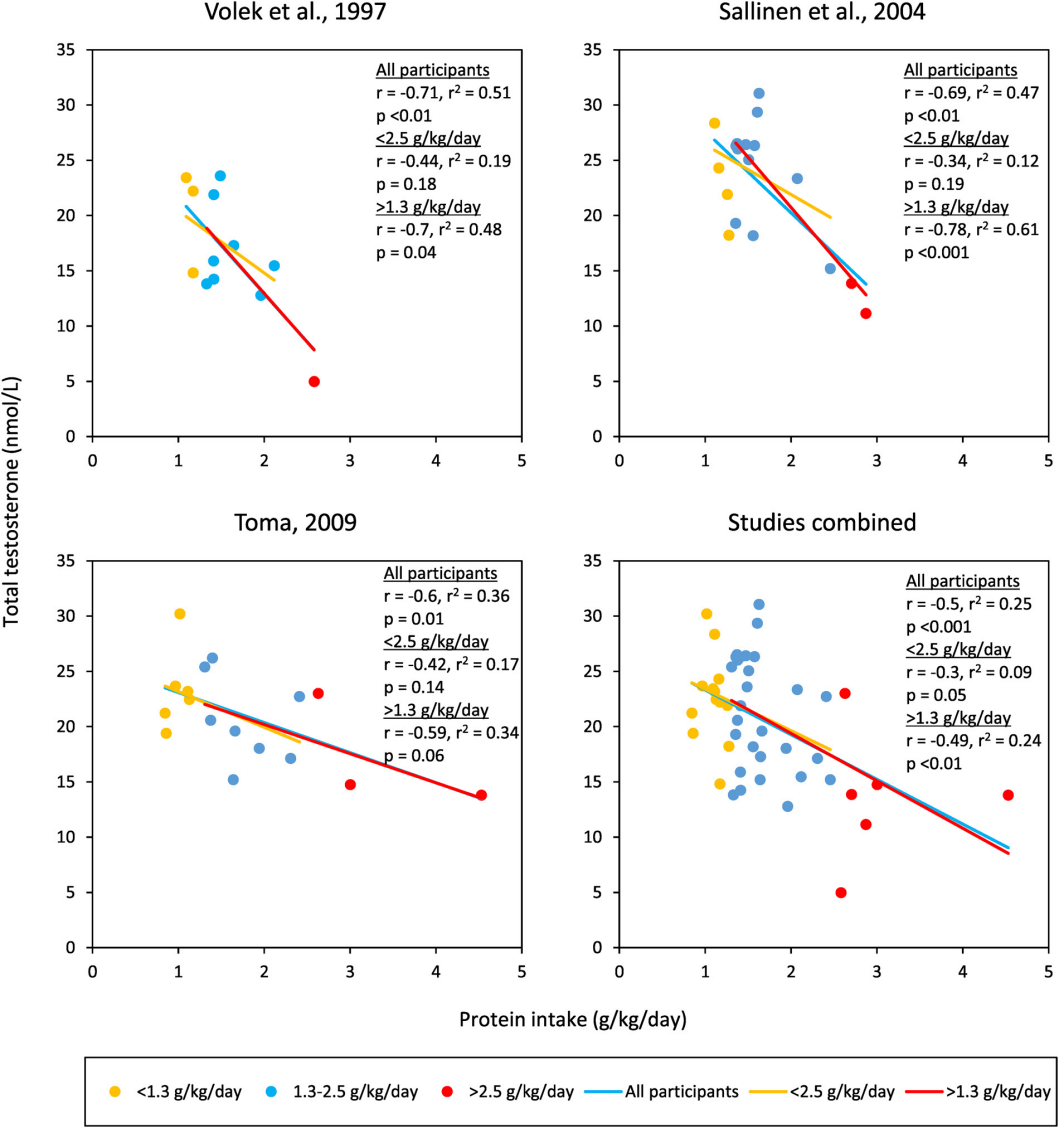
Observational data (*n* = 47) showing an inverse correlation between protein intake and total testosterone, which weakens when participants > 2.5 g/kg/day of protein are removed. Volek et al. (1997) protein intake converted from the percentage of total energy intake. All data extracted from graphs using WebPlotDigitizer (Rohatgi, 2022), incurring very minor differences from the original data. Volek et al. (1997) and Sallinen et al. (2004): serum testosterone; Toma (2009): plasma testosterone. g/kg/day: g/kg of bodyweight/day.

Large observational studies have found no correlation between protein intake and TT (Allen et al., 2002; Field et al., 1994); however small observational studies in athletic populations have found an inverse correlation (*r* = ∼−0.7) (Sallinen et al., 2004; Volek et al., 1997). Also, Toma (2009) found an inverse correlation between protein intake and TT (*r* = −0.6), within an intervention study. Both the small observational studies and Toma (2009) included participants with a wide range of protein intakes, owning to an athletic population in the former, and a dietary intervention in the latter. In contrast, the larger observational studies using the general population, likely had a tighter and lower distribution of protein intakes, with proportionally fewer participants > 2.5 g/kg/day protein. Figure 1 shows the individual participant data of the two small observational studies and Toma (2009). The inverse correlation between protein intake and TT substantially weakens after excluding participants > 2.5 g/kg/day protein, whereas excluding participants < 1.3 g/kg/day has little effect. This indicates that whilst going from 1.3–2.5 to > 2.5 g/kg/day is associated with a decrease in TT, going from < 1.3 to 1.3–2.5 is not associated with a decrease in TT. In other words, the association between lower TT and higher protein is only found in diets > 2.5 g/kg/day. This supports the findings of the aforementioned intervention studies, which show only diets > 3.4 g/kg/day of protein cause a consistent decrease in TT. It also suggests diets 2.5–3.4 g/kg/day may decrease TT, although intervention studies within that range are conflicting (Table 2).

### Mechanisms

The most obvious mechanism for the decrease in TT on diets > 3.4 g/kg/day protein revolves around the urea cycle, as diets > 3.35 g/kg/day may surpass the MRUS. Rodents fed protein 60% of TEI have shown the ability to upregulate the urea cycle and increase their MRUS; which is partly achieved via hormonal regulation (Rémésy et al., 1988). In humans, testosterone replacement therapy in hypogonadal men decreases hepatic urea production and protein loss, indicating higher levels of testosterone suppress the urea cycle (Lam et al., 2017). Therefore, the observed decrease in TT on diets > 3.4 g/kg/day, may serve to upregulate the urea cycle. In addition, two studies on diets > 3.4 g/kg/day found a significant increase in cortisol, and one found a non-significant increase (Anderson et al., 1987; Langfort et al., 1996; Lima-Silva et al., 2011). Human and rodent studies indicate that cortisol upregulates the urea cycle (Okun et al., 2015). Therefore, the increase in cortisol on diets > 3.4 g/kg/day protein, may further serve to upregulate the urea cycle. The finding that exogenous cortisol administration and endogenous stimulation decreases TT (Cumming et al., 1983), also suggests that any hormonal effort to upregulate the urea cycle would involve an increase in cortisol, and a reciprocal decrease in testosterone.

To what extent humans have the ability to upregulate the urea cycle or otherwise alter nitrogen metabolism in response to diets > 3.4 g/kg/day protein, is currently unknown, as to date no studies have directly examined this. The aforementioned 4.4 g/kg/day study incurred a high dropout rate, however, the majority of the participants seemingly tolerated the diet; although side effects were not examined in detail (Antonio et al., 2014). This indicates that individuals who tolerated the diet either had high pre-existing MRUS, upregulated their MRUS, or altered nitrogen metabolism via other means (e.g. reduced gastrointestinal absorption). Although alternative or complimentary mechanisms may be at play, the proposed urea cycle-related mechanism simultaneously explains why diets > 3.4 g/kg/day protein consistently decrease TT (to upregulate the urea cycle); whilst diets < 3.4 g/kg/day do not consistently decrease TT (as there is no need to upregulate the urea cycle). However, it should be mentioned that this is mostly theoretical at this point, as there are no direct studies examining this.

Another possibility is that lower TT is a consequence of hyperammonaemia, rather than a response to it. Rodents fed protein 52% of TEI show kidney injury and increased systemic inflammation (Snelson et al., 2021); and fed protein 44% of TEI show increased markers of oxidative stress (Żebrowska et al., 2019). Animal models of induced hyperammonaemia increase systemic inflammation (Balzano et al., 2020); and although not completely consistent, generally increase oxidative stress as well (Bosoi et al., 2012; Yan et al., 2021). The former is supported by clinical data, showing conditions that incur hyperammonaemia via liver cirrhosis, are associated with elevated markers of inflammation (Felipo et al., 2012). Thus, diets >3.4 g/kg/day of protein may induce hyperammonaemia, leading to increased inflammation and oxidative stress. Observational studies show an inverse relationship between testosterone and inflammatory markers (Bianchi, 2018); and endotoxin-induced inflammation has been shown to acutely decrease TT in men (Tremellen et al., 2018); indicating increased inflammation impairs testosterone synthesis. Moreover, hypogonadism is associated with increased oxidative stress, which partly improves upon treatment (Unluhizarci et al., 2020); suggesting oxidative stress decreases testosterone. Thus, inflammation and oxidative stress caused by hyperammonaemia may impair testosterone levels. Whether the increase in blood ammonia on diets > 3.4 g/kg/day protein is sufficient to induce these effects is debatable. It is more likely the decrease in TT on diets > 3.4 g/kg/day protein is a hormonal response to upregulate the urea cycle and increase nitrogen excretion, rather than a consequence of hyperammonaemia.

## Conclusions

Whittaker and Harris’ (2022) meta-analysis found that whilst low-carbohydrate diets > 3.4 g/kg/day protein decreased TT, low-carbohydrate diets < 3.4 g/kg/day protein had no consistent effect on TT. A re-examination of the relevant studies within the meta-analysis, and the wider literature, indicates that diets > 3.4 g/kg/day protein decrease TT, but diets < 3.4 g/kg/day do not; and that this effect is driven by protein intake, rather than carbohydrate or fat. The observed decrease in TT on diets > 3.4 g/kg/day protein, may be part of the hormonal response to upregulate the urea cycle, thereby limiting the adverse effects of hyperammonaemia. The lack of definition around the term ‘high-protein diet’, and the absence of context whilst discussing the findings, has led to confusion around the issue of protein intake and testosterone. To clarify, only very high-protein diets (> 3.4 g/kg/day) show a consistent decrease in TT, high- and moderate-protein diets (1.25–3.4 g/kg/day) do not. To put this into context, the general population's protein intake is ∼1.3 g/kg/day, conventional ‘high-protein diets’ rarely surpass ∼3 g/kg/day, and the vast majority of athletes are < 3.4 g/kg/day (Fulgoni, 2008; Jenner et al., 2019). The effects of 2.5–3.4 g/kg/day protein on testosterone are less clear, and future research should aim to elucidate these. Moreover, as exercise increases protein requirements, it may curtail any negative effect of > 2.5 g/kg/day protein on testosterone, and ought to be explored. Different protein sources may have differential effects on TT, thus future research may also examine the effects of plant versus animal, and whole food versus powdered protein. Lastly, whether the adverse effects on TT of diets >3.4 g/kg/day protein continue long-term (> 2 weeks) is important to elucidate.

## Abbreviations

g/kg of bodyweight/day: g/kg/day
MRUS: maximal rate of urea synthesis
TEI: total energy intake
TT: total testosterone

## Notes

1. The original study is slightly unclear, in that it implies all 10 drop-outs were from the treatment group, but does not explicitly state this.
